# Smokeless tobacco utilization among tribal communities in India: A population-based cross-sectional analysis of the Global Adult Tobacco Survey, 2016–2017

**DOI:** 10.3389/fpubh.2023.1135143

**Published:** 2023-03-09

**Authors:** Ritik Agrawal, Shishirendu Ghosal, Jogesh Murmu, Abhinav Sinha, Harpreet Kaur, Srikanta Kanungo, Sanghamitra Pati

**Affiliations:** ^1^Division of Public Health, ICMR-Regional Medical Research Centre, Bhubaneshwar, Odisha, India; ^2^Department of Health Research, Health Technology Assessment in India (HTAIn), ICMR-Regional Medical Research Centre, Bhubaneshwar, Odisha, India; ^3^Division of Epidemiology and Communicable Disease, Indian Council of Medical Research, New Delhi, India

**Keywords:** tobacco, GATS-2, ethnicity, smokeless tobacco, India, tribes, khaini, SLT

## Abstract

**Introduction:**

Evidence on smokeless tobacco use is scarce among indigenous communities, with the available literature based either on a specific tribe or on a particular region. Therefore, we aimed to estimate the prevalence of smokeless tobacco and assess its correlation among tribal communities in India.

**Methods:**

We utilized data from the Global Adult Tobacco Survey-2 conducted in 2016–2017. A total of 12,854 tribal people aged >15 years were included in this study. The utilization of smokeless tobacco was estimated using the weighted proportion, and its correlates were assessed by multivariable logistic regression reported as an adjusted odds ratio (AOR) with a 95% confidence interval.

**Results:**

The prevalence of smokeless tobacco use was 32%. Participants aged 31–45 years [AOR: 1.66 (1.37–2.00)], who were men [AOR: 2.37 (1.94–1.90)], and who were daily wage/casual laborers [AOR: 5.32 (3.39–8.34)] were observed to have a significant association with smokeless tobacco. Willingness and attempt to quit smokeless tobacco were higher in Eastern India (31.2%) and central India (33.6%), respectively.

**Discussion:**

We observed one-third of the tribal individuals used smokeless tobacco in India. Tobacco control policies should prioritize men, rural residents, and individuals with fewer years of schooling. Culturally appropriate and linguistically tailored messages are required for behavioral change communication.

## Introduction

The tobacco products used without combustion through chewing, snuffing, spitting, applying on teeth and gums, and dipping are known as smokeless tobacco (SLT) (1). The most commonly used SLT products in India are khaini (a mixture of dried tobacco with lime), gutkha, paan masala (flavored/sweetened areca nut mixed with tobacco), mawa, betel quid with tobacco, sada/surti, nassi/snuff, and other items, such as bajjar, mishri, gudakhu, and gul (2). SLT contains nicotine, which is easily absorbed through the mucosal membrane and is highly addictive in nature (3). Globally, approximately 248 million people use SLT, of which 90% live in India where tobacco-related morbidity and mortality remain a major public health challenge (4). According to the GATS (2016–2017) India survey report, approximately 20 million adults (21.4%) frequently consumed one or the other type of SLT (2). Tobacco consumption can cause oral potentially malignant disorders (OPMDs) such as leukoplakia, erythroplakia, tobacco pouch keratosis, and oral submucous fibrosis (5). In addition, SLT is also associated with adverse effects on physical health, which include myocardial infarction, obstructive pulmonary diseases, infertility, and cancer of various organs such as the throat, oral cavity (6), esophagus, and pancreas (7). A systematic review conducted to summarize the risks of tobacco use found a substantial risk of oral or oropharyngeal cancers to be associated with SLT use in India (8).

The prevalence of SLT consumption (21.4%) is double that of smoking (10.7%) in India, which necessitates the need for investigating the burden of smokeless tobacco and its determinants (9). Furthermore, the use of SLT increases among the tribal population which could be associated with their sociocultural practices including easy availability and wider acceptability in society (2, 5). According to the 2011 Census of India, scheduled tribes (STs) accounted approximately 104 million in population, which is nearly 8.6% of the national population (6), belonging to 505 tribes including 75 particularly vulnerable tribal groups (PVTGs) (8). Indigenous populations live mostly in rural areas and apparently carry distinct cultural and traditional values which keep them apart from mainstream socioeconomic activities (9). SLT use is typically initiated at an early age and continues throughout life, which is often conveyed from generation to generation (10, 11). A study conducted in India found the prevalence of SLT use to be approximately 63.4% among tribal (12).

Although existing tobacco control programs such as the National Tobacco Control Programme (NTCP) have helped in reducing the SLT burden, there is a need to monitor the current status of tobacco use among tribal communities since they are more vulnerable to its use. The majority of the evidence on SLT use among tribes stems from a localized region or is based on a survey of a specific tribe, with no nationally representative study. Keeping in view the vast tribal population of India and their distinct health-related behaviors, it is critical to develop evidence on SLT use in this community. Hence, we aimed to estimate the prevalence of smokeless tobacco including different types of SLTs, and determine its correlates and the regional variations among tribal people in India using nationally representative data from GATS-2, 2016–2017.

## Methods

### Overview of data

We employed data from the second edition of the Global Adult Tobacco Survey (GATS) conducted during 2016–2017 in India. GATS is a nationwide cross-sectional survey of a representative adult population aged ≥15 years. It employed a standard methodology to collect community-based data on tobacco use and its control indicators. Approximately 74,037 participants were surveyed during GATS 2 across 30 states and two union territories of India using a multi-stage cluster sampling method. To reach the final unit of sampling, three-stage and two-stage sampling designs were employed in urban and rural areas, respectively.

The eligibility of each household member was determined by administering the household questionnaire. One adult was randomly chosen from a list of all eligible adult occupants of the household to complete an individual questionnaire. The detailed methodology for GATS-2 can be referred to elsewhere (13).

### Study participants and sample size

In this study, we included tribal people aged ≥15 years who were interviewed during the GATS-2 survey. GATS-2 enquired about the ethnicity of the participants based on the question “Do you belong to a scheduled caste, scheduled tribe, other backward castes, or none of these groups?” The participants who responded their caste to as “scheduled tribe” were included in this study. Out of 74,037 individuals surveyed, 12,854 participants belonged to the scheduled tribe (ST) community forming the ultimate sample size.

### Outcome variable

The main variable of interest was SLT users, which were formed on the basis of the question “Do you currently use smokeless tobacco on a daily basis, less than daily, or not at all?” Those who responded to their SLT use status to be “daily” or “less than daily” were considered SLT users, while those who answered as “not at all” were grouped as SLT non-users.

### Independent variables

We classified age, a continuous variable, into four categories: 15–30 years, 31–45 years, 46–60 years, and ≥61 years. Gender was recorded as male and female. The residence of the participants was grouped as rural and urban. Based on the geographical location, Indian states were grouped into six regions, namely, east, west, north, south, central, and northeast. Education of the respondents was classified into five groups, namely, no formal schooling, primary incomplete, primary but not secondary, secondary and higher, and graduation and above. Occupation of the participants described their current working status with groups being government and non-government employees, self-employed, unemployed, homemaker, daily wage/casual laborer, and student. The wealth index was calculated by reducing the dimensions of 14 household assets employing principal component analysis (PCA). The scores obtained were stratified into 5 quintiles with the lowest score as denoting poverty, the highest score denoting wealth, and the middle three scores clubbed together as the middle class. Smoking status was grouped as smokers and non-smokers. Participants who responded that they want to quit SLT were termed as willing to quit, while those who tried to quit tobacco in the last 12 months were grouped as ‘attempted to quit'. A detailed description of each variable is provided in Supplementary material S1.

### Statistical analysis

STATA version 17.0 (STATA Corp., Texas) was used for analysis. We used mean and standard deviation to report the age of participants along with the age of SLT initiation. The prevalence of SLT among various sociodemographic characteristics was reported as frequency and proportion (%). Patterns of various SLT consumption were estimated through a simple matrix approach, which was presented for all possible combinations of SLT products (14). The GATS sampling weights were used to justify the differential probabilities of participant selection. All weighted proportions were reported along with 95% confidence intervals (CI) as a measure of uncertainty. A binary logistic regression was performed to assess the association between SLT and various sociodemographic characteristics presented as an odds ratio (OR) with 95% CI. A multivariable logistic regression assessed the determinants of SLT use reported as an adjusted odds ratio (AOR) with 95% CI. Variables with a *p* < 0.05 were considered statistically significant. Willingness and attempt to quit tobacco across various regions of India were also presented as frequency and proportion.

### Ethical considerations

There is no risk to the participants because the current study is based on secondary, anonymized GATS-2 data. The original GATS survey administered informed written consent to all the respondents before participation.

## Results

The mean age of participants was calculated to be 37.7 (±14.9) years, with more than two-thirds of the population aged between 15 and 45 years. Approximately one-fifth of the population (21.3%) were urban residents, and the gender proportion was marginally skewed toward women (54.38%). The prevalence of SLT use among the tribal population was 32.2% (95% CI: 31.32–33.00). The mean age of initiation of SLT use was observed to be approximately 20.7±8.1 years. We observed that approximately 9% of the SLT users were younger than 21 years of age.

Among the tobacco products, “khaini or tobacco lime mixture” was the most frequently [16.0% (95% CI: 15.4–16.7)] used, followed by “gutkha/tobacco lime and areca nut mixture” [7.3% (95% CI: 6.8–7.8)] and “oral tobacco, snuff, gul, and gudakhu” [7.1% (95% CI: 6.7–7.6)]. Khaini along with gutkha was the most widely used combination [5.55% (95% CI: 4.79–6.37)], followed by khaini and gul [3.71% (95% CI: 3.09–4.41)], among our target population. A prevalence of 1.53% (95% CI: 1.13–2.00) was even accounted for consuming all the major SLT products (i.e., khaini, betel, gutkha, gul, paan, and snuff). A detailed description of commonly used SLT products is presented in Table 1.

**Table 1:** Weighted prevalence of different types of smokeless tobacco use (*n* = 12,128).

**Types of SLT**	***n*, % (95% CI)**
Betel quid with tobacco product	463, 3.82% (3.48–4.17)
Khaini or tobacco lime mixture	1,940, 16.00% (15.35–16.66)
Gutkha /tobacco lime, areca nut mixture	884, 7.29% (6.83–7.77)
Oral tobacco, such as snuff and gul gudakhu	862, 7.11% (6.66–7.58)
Pan masala and betel quid without tobacco	261, 2.15% (1.90–2.43)
Nasal use of snuff	103, 0.85% (0.69–1.03)
Others	56, 0.46% (0.35–0.60)
**Combination of SLT product use (*****n*** = **3,339)**	
Khaini + Gutkha	185, 5.55% (4.79–6.37)
Khaini + Gul	124, 3.71% (3.09–4.41)
Khaini + Betel	55, 1.64% (1.24–2.14)
Khaini + Betel + Gutkha + Gul + Paan + Snuff	51, 1.53% (1.13–2.00)
Gul + Gutkha	43, 1.27% (0.93–1.73)
Khaini + Paan	42, 1.26% (0.91–1.70)
Gul + Gutkha + Khaini	40, 1.20% (0.86–1.63)

The highest prevalence of SLT use was recorded among the 31–45 years age group [39.7% (95% CI: 38.1–41.4)]. Smokeless tobacco use was higher among men (41.63%) than women (22.84%). We observed a significant proportion of the participants with no formal education [35.5% (95% CI: 34.0–36.9)], incomplete primary education [38.7% (95% CI: 36.1–41.4)], and incomplete secondary education [37.6% (95% CI: 36.0–39.3)] were smokeless tobacco users. Smokeless tobacco use was most prevalent among the tribal people from the eastern part [42.3% (95% CI: 40.5–44.0)] of India. Smokeless tobacco use in other regions, such as Northeast India [35.0% (95% CI: 32.4–37.5)] and central India [36.2% (95% CI: 34.6–37.9)], was also high. With an increase in the wealth index, a gradual decrease in the use of SLT products was observed (Table 2).

**Table 2:** Distribution of sociodemographic characteristics across SLT users in GATS-2.

**Socio-demographic characteristics**	**Categories**	**Total n, %**	**SLT user; *n*, % (95% CI)**
Age (years) (*n* = 12,128)	15–30	5,396, 44.50%	1,324, 24.5% (23.4–25.7)
	31–45	3,506, 28.91%	1,393, 39.7% (38.1–41.4)
	46–60	2,118, 17.46%	790, 37.3% (35.2–39.4)
	>60	1,108, 9.13%	392, 35.4% (32.6–38.3)
Gender (*n* = 12,128)	Male	6,007, 49.53%	2,501, 41.63% (40.4–42.9)
	Female	6,121, 50.47%	1,398, 22.84% (21.8–23.9)
Residence (*n =* 12,128)	Urban	2,013, 16.60%	502, 24.9% (23.0–26.8)
	Rural	10,115, 83.40%	3,397, 33.6% (32.6–34.5)
Region (*n =* 12,128)	North	182, 1.50%	8, 4.4% (1.9–8.5)
	Central	3,324, 27.41%	1,204, 36.2% (34.6–37.9)
	East	3,203, 26.41%	1,354, 42.3% (40.5–44.0)
	Northeast	1,384, 11.41%	484, 35.0% (32.4–37.5)
	West	2,139, 17.63%	550, 25.7% (23.9–27.6)
	South	1,896, 15.63%	299, 15.8% (14.1–17.5)
Educational qualification (*n =* 12,114)	No formal education	4,506, 37.19%	1,598, 35.5% (34.0–36.9)
	Primary incomplete	1,353, 11.17%	524, 38.7% (36.1–41.4)
	Primary but not secondary	3,379, 27.89%	1,271, 37.6% (36.0–39.3)
	Secondary and higher secondary	2,218, 18.31%	419, 18.9% (17.3–20.6)
	Graduation and above	658, 5.43%	82, 12.4% (10.0–15.2)
Occupation (*n =* 12,120)	Govt. and NON–GOVT. EMployee	839, 6.92%	246, 29.3% (26.2–32.5)
	Daily wage/Casual laborer	3,992, 32.93%	1,726, 43.2% (41.7–44.8)
	Self–employed	2,410, 19.89%	1,005, 41.7% (39.7–43.7)
	Homemaker	3,042, 25.10%	584, 19.2% (17.8–20.6)
	Unemployed	712, 5.88%	242, 34.0% (30.5–37.6)
	Student	1,125, 9.28%	96, 8.5% (6.9–10.3)
Wealth index (*n =* 12,020)	Poor class	4,692, 39.03%	1,663, 35.4% (34.1–36.8)
	Middle class	5,956, 49.55%	1,929, 32.4% (31.2–33.6)
	Rich class	1372, 11.41%	269, 19.7% (17.5–21.8)
Smoking tobacco (*n =* 12,128)	Yes	1,542, 12.71%	536, 34.8% (32.4–37.2)
	No	10,586, 87.29%	3,363, 31.8% (30.9–32.7)

The highest chances of SLT use were observed to be among participants aged 31–45 years [AOR: 1.66 (1.37–2.00)] as compared with the respondents aged 15–30 years (Table 3). Male participants had 2.37 times higher odds [AOR: 2.37 (1.94–1.90)] of consuming SLT than their female counterparts. The northeastern tribal population had a higher likelihood of using SLT [AOR: 4.88 (3.66–6.50)] than the southern population. Daily wage/casual laborers had 5.32 times higher odds [AOR: 5.32 (3.39–8.34)] of being smokeless tobacco users in comparison to the students. The deprived group had 48% higher chances of being smokeless tobacco users [AOR: 1.48 (1.06–2.07)] as compared with the wealthiest group.

**Table 3:** Univariable and multivariable logistic regression models for the association between SLT use and sociodemographic characteristics.

**Socio-demographic characteristics**	**Categories**	**Odds ratio (95% CI)**	**AOR (95% CI)**
Age	15–30 years	Reference	
	31–45 years	2.03 (1.71–2.40)	1.66 (1.37–2.00)
	46–60 years	1.82 (1.49–2.24)	1.41 (1.12–1.78)
	>60 years	1.68 (1.32–2.15)	1.45 (1.08–1.93)
Gender	Male	1.36 (1.26–1.46)	2.37 (1.94–1.90)
	Female	Reference	
Residence	Urban	Reference	
	Rural	1.52 (1.20–1.92)	0.94 (0.73–1.22)
Region	North	0.26 (0.12–0.58)	0.46 (0.21–0.99)
	Central	3.03 (2.29–4.02)	3.50 (2.61–4.70)
	East	3.91 (2.94–5.20)	4.71 (3.51–6.32)
	Northeast	2.87 (2.20–3.74)	4.88 (3.66–6.50)
	West	1.85 (1.34–2.55)	2.24 (1.61–3.13)
	South	Reference	
Educational qualification	No formal education	3.88 (2.62–5.74)	3.25 (2.02–5.23)
	Primary incomplete	4.45 (2.94–6.76)	2.82 (1.73–4.59)
	Primary but not secondary	4.25 (2.85–6.35)	3.35 (2.10–5.34)
	Secondary and higher secondary	1.64 (1.06–2.54)	1.61 (0.99–2.64)
	Graduation and above	Reference	
Occupation	Govt. and non–govt. employee	4.45 (2.71–7.31)	4.14 (2.41–7.10)
	Daily wage/casual laborer	8.18 (5.48–12.21)	5.32 (3.39–8.34)
	Self–employed	7.68 (5.12–11.54)	4.84 (3.09–7.59)
	Homemaker	2.55 (1.69–3.85)	2.44 (1.54–3.86)
	Unemployed	5.53 (3.47–8.82)	3.92 (2.36–6.52)
	Student	Reference	
Wealth index	Poor class	2.24 (1.70–2.96)	1.48 (1.06–2.07)
	Middle class	1.96 (1.49–2.57)	1.33 (0.97–1.84)
	Rich class	Reference	
Smoking tobacco	Yes	1.14 (0.95–1.38)	0.59 (0.47–0.75)
	No	Reference	

We found a high proportion of SLT users from central India (30.1%) and Eastern India (31.2%) were willing to quit tobacco use. In addition, a higher proportion of tribal people who attempted to quit SLT belonged to the central (33.6%) and eastern (31.6%) regions of India (Table 4).

**Table 4:** Regional differences between willingness to quit tobacco and attempted to quit tobacco.

**Region**	**Willingness to quit (*N =* 1,869)**	**Attempted to quit (*N =* 1,031)**
	***n*** **, % (95% CI)**	***n*** **, % (95% CI)**
**North**	7, 0.39% (0.15–0.77)	5, 0.52%, (0.16–1.13)
**Central**	563, 30.13%, (28.04–32.26)	346, 33.57%, (30.68–36.53)
**East**	583, 31.19%, (29.10–33.35)	326, 31.61%, (28.79–34.56)
**Northeast**	277, 14.83%, (13.24–16.51)	107, 10.42%, (8.58–12.40)
**West**	244, 13.05%, (11.56–14.67)	101, 9.78%, (8.05–11.78)
**South**	195, 10.41%, (9.08–11.91)	146, 14.12%, (12.00–16.34)

## Discussion

We observed the overall prevalence of SLT to be high. Respondents of middle age, male, northeastern region, fewer years of education, daily wage/casual laborers, and poor wealth quintile were associated with smokeless tobacco consumption among tribes. In the eastern region, people were more willing to quit SLT, whereas a higher proportion of respondents from central India attempted to quit SLT.

In the current study, the mean age of initiation of SLT was 20.7 (±8.0) years. However, our findings are slightly higher than a study conducted among the tribal community of West Bengal, which found the mean age of initiation of SLT to be ~17.5 years (15). In addition, based on the GATS-2 data, we observed the mean age of initiation of SLT in the general population was lower (18.8 years) than that reported among tribes in the present study (2). This suggests that there is late initiation of SLT use among tribes, which can be a window of opportunity for behavioral change communication to persuade them to refrain from tobacco consumption.

This study highlighted the overall prevalence of SLT use among tribal to be approximately 32% which is in harmony with the results of a similar study conducted among urban tribes in Madhya Pradesh that found the prevalence of SLT to be approximately 37.2% (16). However, a study conducted among the “Mishing” tribe of Assam showed a higher prevalence (48.5%) of SLT than our study (17). The probable reason for this difference could be regional and socio-cultural characteristics. Here, it is worth noting that the SLT consumption among tribes is much greater than that of the general population (21.4%) in India, as reported by GATS-2 (18). A study conducted by Thakur et al. (15) revealed that khaini was the most commonly used form of SLT (46.5%) which is similar to the results of the present study (15).

The current study suggests that the prevalence of SLT was highest among participants aged 31–45 years, which adheres to the findings of a study conducted among the rural tribes of Nagaland where the use of SLT was highest among respondents aged 25–44 years (19). This highlights that the existing tobacco control policies should focus more on the younger age group, which could help in limiting its use. We observed a decline in SLT consumption with an increase in age, which is consistent with the findings among rural tribes of Nagaland, which highlighted a uniform reduction in SLT use among both men and women between 25–44 years and 45–64 years of age (19). The above results indicate a decline in tobacco use among the middle and older aged population indicating the need for more attention toward the younger age group. Furthermore, we found male participants had a higher likelihood of using tobacco than their female counterparts, which is similar to a study conducted among the “Narikurava” community in Tamil Nadu (12).

We also observed an association between SLT consumption and lower educational attainment. Our result is in harmony with the observations of a study from Madhya Pradesh, which showed tribal individuals with primary education rampantly used SLT (20). This signifies the importance of education for better implementation and effectiveness of the existing tobacco control programs for this vulnerable group. Participants from the poorest wealth quintiles were at a higher risk of SLT use which is similar to a study conducted among the “Sugali” tribe of Telangana (21). In addition, a review suggested that the SLT is consumed more among the poorest wealth quintiles of the general population (22). For the year 2017–2018, the total economic costs attributed to SLT consumption among the general population aged ≥35 years for all diseases and deaths constituted to be approximately INR 1773.4 billion of which SLT accounts for nearly 26% (23).

In the present study, the northeastern region was found to be the highest utilizer of smokeless tobacco, followed by the eastern region. This is similar to the findings of NFHS-5 (2019–2021), a nationally representative survey, which suggested that the prevalence of SLT consumption among the general population was higher in the northeastern states (24). The substantially widespread societal acceptance of SLT consumption and cultural norms, greater availability, and easy access to tobacco products were likely to be key contributing factors to increased SLT use in the northeastern area (25–27). In addition, enculturation, parental influence, peer pressure, workplace availability, marginalization, and perceived health benefits also induce tobacco use (11).

We observed a higher proportion of people from Eastern India and central India were willing to quit SLT and have attempted to do so, which indicates that these people can easily be persuaded to quit tobacco. Tailored anti-tobacco messages in the local language may prove to be beneficial. Personal support, if required, may be extended by program implementers, which will aid in quitting tobacco. Tobacco taxes are a proven strategy for reducing tobacco consumption and also restraining the availability of these products (28). The National Tobacco Control Programme (NTCP) and Prohibition of Advertisement and Regulation of Trade and Commerce, Production, Supply, and Distribution Act, and COTPA, 2003, have been effective in reducing the burden of tobacco in India (29). In 2003, World Health Organization (WHO) initiated the Framework Convention on Tobacco Control (WHO-FCTC). Article 6 of the FCTC calls for the imposition of taxes on tobacco products as a credible tool to decrease its demand (30). However, a wide gap still exists in SLT use between the tribes and the general population.

Findings from the current study reflect that the prevalence of SLT consumption is much higher among tribes than the general population, which calls for urgent action in the tribal-dominated regions. The existing program should be implemented in such a way that it should be culturally acceptable as well as linguistically understandable among these groups to create a positive effect. Though the prevalence of SLT use was high among men, SLT use is reasonably rising among women than smoking, which poses equivalent health hazards (29).

### Implications

The consumption of SLT products was relatively greater than the general/non-tribal population. The use of SLT was more among tribal individuals with fewer years of schooling, who should be focused while planning for health awareness, tobacco cessation activities, or behavior change communication (BCC). Mass media advertisements or camps could be conducted, where visual or graphical presentations must be a part of awareness, which can be beneficial for all segments of society. The involvement of tribal leaders and celebrities in anti-tobacco campaigns in their local language may arouse interest in the subject. Folk dances, puppet shows, and other traditionally acceptable activities can be implemented. Special attention to tobacco cessation policies is essential for the seven sisters of northeast India, owing to the pervasiveness of the higher burden.

### Strength and limitations

The Global Adult Tobacco Survey (GATS) provides an extensive and nationally representative dataset with better generalizability of the findings. We considered the self-reported cases of SLT use, which may lead to underestimation (misclassification bias and recall bias) of the actual burden of tobacco. The cross-sectional design hampered our abilities to draw causal relationships.

## Conclusion

This study highlights the high burden of tobacco use along with its social determinants, which can help in tailoring the tobacco control program among tribes in India. Tobacco control programs should target men, rural residents, and individuals with fewer years of schooling. Future studies should explore the behavioral and social linkages to tobacco use among tribal communities.

## Data availability statement

The original contributions presented in the study are included in the article/Supplementary material, further inquiries can be directed to the corresponding authors.

## Author contributions

SG, AS, SK, and SP: conceptualization and methodology. AS and SP: data curation. RA, JM, and SG: formal analysis. SK and SP: software and supervision. RA, JM, SG, and AS: writing the original draft. SP, HK, and SK: writing, reviewing, and editing. All authors contributed to the article and approved the submitted version.
