# Supplementary material for: Smokeless tobacco utilization among tribal communities in India: A population-based cross-sectional analysis of the Global Adult Tobacco Survey, 2016–2017
Source: Front Public Health. 2023 Mar 9;11:1135143. doi: 10.3389/fpubh.2023.1135143 (PMC10034193; doi:10.3389/fpubh.2023.1135143)
Supplement: Supplementary file 1 [file Table_1.pdf]

Supplementary Table S1: Description of variables in the study, Global Adult Tobacco Survey (GATS-2), 2017-18		
**Variables**	**Question**	**Categories**
**Age**	What is the age of the selected person? (Age in completed years)	Recoded into:<br>1. 15-30 years<br>2. 31–45 years<br>3. 46-60 years<br>3. ≥ 60 years
**Gender**	The gender was recorded based on the observation	1.Male<br>2. Female
**Residence**	Based on the residence status	1.Urban<br>2. Rural
**Region**	Based on the national region	1.North<br>2. Central<br>3. East<br>4. North East<br>5. West<br>6. South
**Educational Qualification**	What is the highest level of education you have completed?	Recoded into:<br>1=1 No formal education (No formal schooling)<br>2=2 Primary Incomplete (Less than primary school completed)<br>3/4=3 Primary but not Secondary (Primary school completed + Less than secondary school completed)<br>5/6=4 Secondary and Higher Secondary (Secondary school completed + Higher secondary school completed)<br>7/8=5 "Graduation and Above (College/university completed + Post graduate degree completed)
**Occupation**	Which of the following best describes your work status over the past 12 months?	Recoded into:<br>1/2=1 Govt. and Non-Govt. employee (Government employee + non-government employee)<br>3=2 Daily wage (Daily wage/ casual labourer)<br>4=3 Self-employed (Self-employed)<br>5=4 Student (Student)<br>6=5 Homemaker (Homemaker)<br>7/9=6 Unemployed (Unemployed, able to work + Unemployed, unable to work)
**Wealth Index**	PCA of 14 household assets	Recoded into:<br>1.Poor class<br>2. Middle class<br>3. Rich class

**Smoking tobacco**	Do you currently smoke tobacco on a daily basis, less than daily or not at all?	Recoded into:<br>1/2=1 Smoker (Daily + Less than daily)<br>3=2 Non-smoker (Not at all)
**Smokeless tobacco**	Do you currently use smokeless tobacco on a daily basis, less than daily or not at all?	Recoded into:<br>1/2=1 Smokeless tobacco user (Daily + Less than daily)<br>3=2 Non-user (Not at all)
**Betel**	On average, how many times a day do you use the following products? Also, let me know if you use the product, but not every day. Betel quid with tobacco?	Recoded into:<br>(0=0) (999=0) non-user<br>1/888=1 Betel quid user
**Khaini**	On average, how many times a day do you use the following products? Also, let me know if you use the product, but not every day) Khaini or tobacco lime mixture.	Recoded into:<br>(0=0) (999=0) non-user<br>1/888=1 Khaini user
**Gutkha**	On average, how many times a day do you use the following products? Also, let me know if you use the product, but not every day.) Gutka, areca nut-tobacco lime mixture, or mawa?	Recoded into:<br>(0=0) (999=0) non-user<br>1/888=1 Gutkha user
**Gul**	On average, how many times a day do you use the following products? Also, let me know if you use the product, but not every day.) Oral tobacco use (as mishri, qul, gudakhu)?	Recoded into:<br>(0=0) (999=0) non-user<br>1/888=1 Gul user
**Pan**	On average, how many times a day do you use the following products? Also, let me know if you use the product, but not every day.) Pan masala together with tobacco?	Recoded into:<br>(0=0) (999=0) non-user<br>1/888=1 Pan user
**Snuff**	On average, how many times a day do you use the following products? Also, let me know if you use the product, but not every day.) Nasal use of snuff?	Recoded into:<br>(0=0) (999=0) non-user<br>1/888=1 Snuff user
**Others**	On average, how many times a day do you use the following products? Also, let me know if	Recoded into:<br>(0=0) (999=0) non-user<br>1/888=1 Others

	you use the product, but not every day.) Any others?	
**Willingness to quit**	Which of the following best describes your thinking about quitting smoking?	Recoded into:<br>1/3=1 (Willing to quit)<br>(4=0) (7=0) (Not willing to quit)
**Attempted to quit**	During the past 12 months, did you use any of the following to try to stop smokeless tobacco?	Recoded into:<br>1.Yes<br>2. No
